# Molecular Diversity and Genetic Relatedness of *Candida albicans* Isolates from Birds in Hungary

**DOI:** 10.1007/s11046-021-00527-3

**Published:** 2021-01-29

**Authors:** M. Domán, L. Makrai, Gy. Lengyel, R. Kovács, L. Majoros, K. Bányai

**Affiliations:** 1 Centre for Agricultural Research, Institute for Veterinary Medical Research, P.O. Box 18, Budapest, 1581 Hungary; 2 Department of Microbiology and Infectious Diseases, University of Veterinary Medicine, Budapest, Hungary; 3 Laboratory of Epidemiological Virology, Hungarian Defence Forces Military Medical Centre, Budapest, Hungary; 4 Department of Medical Microbiology, Faculty of Medicine, University of Debrecen, Debrecen, Hungary

**Keywords:** *Candida albicans*, Multilocus sequence typing, Birds, Clade, Genetic diversity

## Abstract

The molecular epidemiology of *Candida albicans* infections in animals has been rarely studied. In this study, multilocus sequence typing was used to characterise the genetic diversity and population structure of 24 avian origin *C. albicans* isolates collected from different birds with candidiasis and compared to human isolates. Fourteen diploid sequence types (DSTs) including six new DSTs were determined. Cluster analysis revealed that isolates grouped into 8 clades. Bird isolates mainly belonged to minor clades and Clade 15 with DST 172 was the most common (11 isolates; 45.8%). The remaining isolates were clustered into Clade 7 (5 isolates; 20.8%), Clade 10 (4 isolates; 16.6%), Clade 8 (2 isolates; 8.3%), Clade 4 (1 isolate; 4.2%) and Clade 16 (1 isolate; 4.2%). Unweighted pair group method with arithmetic averages (UPGMA) and eBURST analyses showed that the genetic construction of avian origin *C. albicans* population is fairly diverse. Although species-specific lineages were not found, some degree of separation in the evolution of bird and human strains could be observed.

## Introduction

Candidiasis is a sporadic fungal disease in livestock. *C. albicans* is the most prevalent fungal commensal of normal human and animal digestive microbiota and an environmental pollutant as well [1]. This yeast is also the major opportunistic pathogen responsible for both superficial and disseminated infections. The excessive use of broad-spectrum antibiotics, hormones and immunosuppressants in recent years contribute to the increasing prevalence of *Candida* infections [2]. In birds, contaminated food, beak abnormalities, tongue injuries, the stress of heavy flight or force-feeding predispose to oral or gastrointestinal candidiasis [3]. Since ingestion of contaminated food or drinking water is the usual route of transmission, contaminated environments (e.g. litter from poultry rearing facilities, areas contaminated with human waste) are potential sources for *Candida* exposure for birds. In addition, candidiasis is a zoonotic disease, therefore pathogens originating from different sources may be transferred to other host species [4].

*C. albicans* is predominantly diploid and displays high degree of genetic diversity across isolates, notably variations in the distribution of heterozygous polymorphisms along the genome. Genotyping strains within a microbial species on the basis of DNA sequences at multiple loci has greatly advanced study of the epidemiology and evolutionary phylogenetic of many fungal pathogens including *C. albicans* [5–8]. At the population level, molecular typing has revealed 19 clades of *C. albicans* strains so far [9, 10]. Some of these clades seem to exhibit geographical enrichment or phenotype specificities, however, no correlation between clade assignment and the ability of strains to cause different forms of infection or host specificity has been established yet [11, 12]. Of note*, C. albicans* is member of the CTG clade that translate the CUG codon as serine rather than leucine. This flexibility of the genetic code along with several other genomic properties such as changes in ploidity, loss of heterozygosity and isochromosome formation contributes to their extraordinary adaptability to colonise a variety of host niches and wide range of natural environments, adapt to diverse selective pressures, as well as escape antifungal drugs [13–15].

Molecular epidemiologic studies have mainly focused on candidiasis in humans, but rarely in animals, thus in-depth investigations on molecular typing and evolutionary relationships are still lacking. To broaden our understanding on the population structure and genetic diversity of *C. albicans* strains in birds, we analysed 30 isolates recovered from animal and human hosts and investigated whether the genotype distribution related to their different source.

## Materials and Methods

### Specimen Collection

A total of 30 *C. albicans* isolates (one isolate from each bird) were examined in this study. Samples obtained from moulard and barbary ducks and geese diagnosed with oesophageal mycosis (*n* = 22), a falcon (*n* = 1), an ostrich (*n* = 1) and human patients (*n* = 6) (Table 1). Samples were plated on Sabouraud dextrose agar supplemented with chloramphenicol and incubated at 35 °C for 48 h. Human isolates were randomly selected and preliminary identified as *C. albicans* by Matrix-assisted laser desorption/ionization time of flight mass spectrometer at the University of Debrecen. All yeast isolates were subjected to molecular characterisation by sequencing of internal transcribed spacer (ITS) region of fungal rDNA [16].

**Table 1:** Origin of *C. albicans* isolates and MLST genotypes involved in the study

Host	Isolate number	Origin	MLST loci							DST	CC
			*AAT1a*	*ACC1*	*ADP1*	*MPIb*	*SYA1*	*VPS13*	*ZWF1b*		
goose	ML-1	Oesophagus	55*	14	4	3	6	45	15	365	3
goose	ML-2	Oesophagus	6	3	37	2	38	116	12	482	16
duck	ML-3	Oesophagus	13	3	4	6	34	20	18	172	43
duck	ML-4	Oesophagus	13	3	4	6	34	20	18	172	43
goose	ML-5	Oesophagus	74	7	16	7	13	19	296	3599	17
goose	Om-1	Oesophagus	74	7	16	7	13	19	296	3599	17
goose	Om-2	Oesophagus	13	3	4	6	34	20	18	172	43
duck	Om-7	Oesophagus	13	3	4	6	34	20	18	172	43
duck	Om-8	Oesophagus	74	7	16	7	13	165	14	3595	17
goose	Om-11	Oesophagus	6	3	37	2	38	46	12	840	16
goose	Om-12	Oesophagus	6	3	37	2	38	46	12	840	16
goose	Om-13	Oesophagus	74	7	16	7	13	19	296	3599	17
goose	Om-14	Oesophagus	6	3	37	2	38	46	12	840	16
duck	Om-16	Oesophagus	70	14	8	4	2	10	8	725	1
duck	Om-17	Oesophagus	55	14	4	3	6	45	15	365	3
duck	Om-18	Oesophagus	13	3	4	6	34	20	18	172	43
goose	Om-19	Oesophagus	13	3	4	6	34	20	18	172	43
goose	Om-29	Oesophagus	13	3	4	6	34	20	18	172	43
goose	Om-31	Oesophagus	13	3	4	6	34	20	18	172	43
duck	Om-34	Oesophagus	13	3	4	6	34	20	18	172	43
duck	Om-36	Oesophagus	13	3	4	6	34	20	18	172	43
duck	Om-42	Oesophagus	13	3	4	6	34	20	18	172	43
falcon	12086	Pharynx	53	31	10	36	83	113	111	1019	13
ostrich	Im-12	Intestine	6	3	37	2	38	50	12	3598	16
human	22491	Decubitus	2	5	5	9	2	6	5	79	0
human	7652	Wound	70	14	8	4	2	3	8	623	1
human	14362	Blood	6	3	37	2	38	50	12	3598	16
human	27700	Cervix	21	114	21	19	30	307	22	3600	S
human	38002	Cervix	105	3	5	2	2	24	5	3597	0
human	43279	Pharynx	70	7	8	4	7	10	22	3596	1

*DST* Diploid sequence type assigned by *C. albicans* MLST database based on allelic profiles (allele number combinations)

*Allele type; *CC* Clonal Complex determined by eBURST; *S* singleton

### Identification of yeasts by ITS sequencing

After culturing isolates for two days, genomic DNA from a single colony of each isolate (containing approx. 1 × 10^6^–5 × 10^6^ yeast cells) was extracted using the Fungi/yeast genomic DNA extraction kit (Favorgen) in accordance with the manufacturer’s instructions. DNA samples were stored at  − 20 °C until analysis. Fungus-specific universal primers ITS1 (5′-TCCGTAGGTGAACCTGCGG-3′) and ITS4 (5′-TCCTCCGCTTATTGATATGC-3′) were used to amplify the entire ITS region [2, 17]. The final PCR mixture volume was 15 μl containing 1 µl fungal DNA, 2 µl 10 × DreamTaq buffer, 0.5 µl dNTP (10 mM), 0.5 µl forward and reverse primers (10 µM each), 0.1 µl DreamTaq DNA polymerase (5 U/µl; Thermo Fisher Scientific) and 10.4 µl distilled water. The condition was set up with an initial denaturation step at 95 °C for 3 min, followed by 40 cycles of 95 °C for 30 s, annealing at 50 °C for 30 s, extension at 72 °C for 1 min, and a final extension step at 72 °C for 10 min. After the electrophoresis in 1% agarose gel stained with GelRed (Biotium), the PCR products were purified using Gel/PCR DNA fragments kit (Geneaid). Amplicons were sequenced on both strands using ITS1 and ITS4 primers with BigDye Terminator v3.1. cycle sequencing kit (Thermo Fisher Scientific) on an ABI Prism 3130 Genetic Analyzer (Applied Biosystems). Sequences were edited and assembled using Mega 6 software (https://www.megasoftware.net/) then species level identification was carried out by the BLAST sequence analysis tool (https://blast.ncbi.nlm.nih.gov/Blast.cgi). Generated nucleotide sequences were deposited in GenBank under the following accession numbers: MT136511-MT136532 and MT478010-MT478017.

### Multilocus Sequence Typing (MLST) of *C. albicans* Isolates

The MLST scheme employed for *C. albicans* genotyping was based on partial amplification and sequencing of seven housekeeping genes (*AAT1a*, *ACC1*, *ADP1*, *MPIb*, *SYA1*, *VPS13* and *ZWF1b*) according to a previously published method [5]. Seven independent PCR amplifications were performed for each isolate. The primer sets and their amplicon lengths were described in detail elsewhere [5, 18]. Experimental conditions used in PCRs and Sanger sequencing were the same as mentioned above.

Heterozygosity was identified by the presence of two peaks at the same polymorphic loci on both strands and the consensus sequences of seven loci of all isolates were defined. For each gene, distinct alleles and diploid sequence types (DSTs) were identified and numbered by comparing the sequences with those available in the *C. albicans* MLST database (https://pubmlst.org/organisms/candida-albicans). Novel alleles and allelic combinations (new DTSs) together with sequence chromatograms were submitted to the central MLST database where new numbers were assigned by the curator.

### Phylogenetic and Population Structure Analysis

Nucleotide sequences were modified as described by Tavanti et al. 2005 to label homozygous and heterozygous sites in order to allow the cluster analysis of diploid sequence data [6]. Relationship among concatenated sequences of the seven loci of each isolate were determined using unweighted pair group method with arithmetic averages (UPGMA) algorithm with p-distance model in Mega 6 software. A bootstrap of 1000 replications was used for the construction [9, 19]. MLST clonal complexes (CC) and their founders were predicted by goeBURST algorithm (http://www.phyloviz.net/goeburst/) to analyse population structure and evolutionary relationships between isolates. A CC was defined to contain at least two DSTs sharing any 6 of the 7 MLST alleles (single-locus variant analysis). DSTs that could not be assigned to any group were called singletons.

## Results

All isolates were identified as *C. albicans* by sequencing the internal transcribed spacer region. Partial DNA sequences of the coding regions of the multiple genetic loci included in MLST scheme were concatenated to generate a dataset of 2883 bp for each examined isolate. A total of 57 different alleles were identified among the 30 genotyped *C. albicans* isolates. The *VPS13* locus generated the most number of alleles (*n* = 12), while *ACC1* locus produced the least (*n* = 6). Overall, 66 nucleotide sites were found to be variable among all sequenced loci. Similarly, the *VPS13* locus produced the highest number of polymorphic sites (*n* = 12), while *ACC1* displayed the lowest (*n* = 5) (Table 2). Among the alleles, three new alleles were determined in *ACC1*, *VPS13* and *ZWF1b* loci, respectively, and each were added to the MLST database.

**Table 2:** Characteristics of the seven examined MLST housekeeping genes

Locus	Sequenced fragment size (bp)	Number of alleles	Number of polymorphic sites	Nucleotide position
*AAT1a*	373	8	7	7, 28, 40, 70, 89, 124, 325
*ACC1*	407	6	5	8, 90, 211, 281, 317
*ADP1*	443	7	11	17, 35, 40, 46, 109, 125, 166, 205, 215, 225, 232
*MPIb*	375	8	11	21, 27, 34, 36, 66, 72, 88, 234, 237, 276, 289
*SYA1*	391	7	9	1, 25, 61, 100, 142, 160, 185, 307, 351
*VPS13*	403	12	12	33, 49, 134, 212, 217, 241, 281, 320, 322, 328, 370, 375
*ZWF1b*	491	9	11	23, 31, 43, 49, 55, 262, 274, 337, 379, 439, 482
Total	2883	57	66	–

**Fig. 1:**
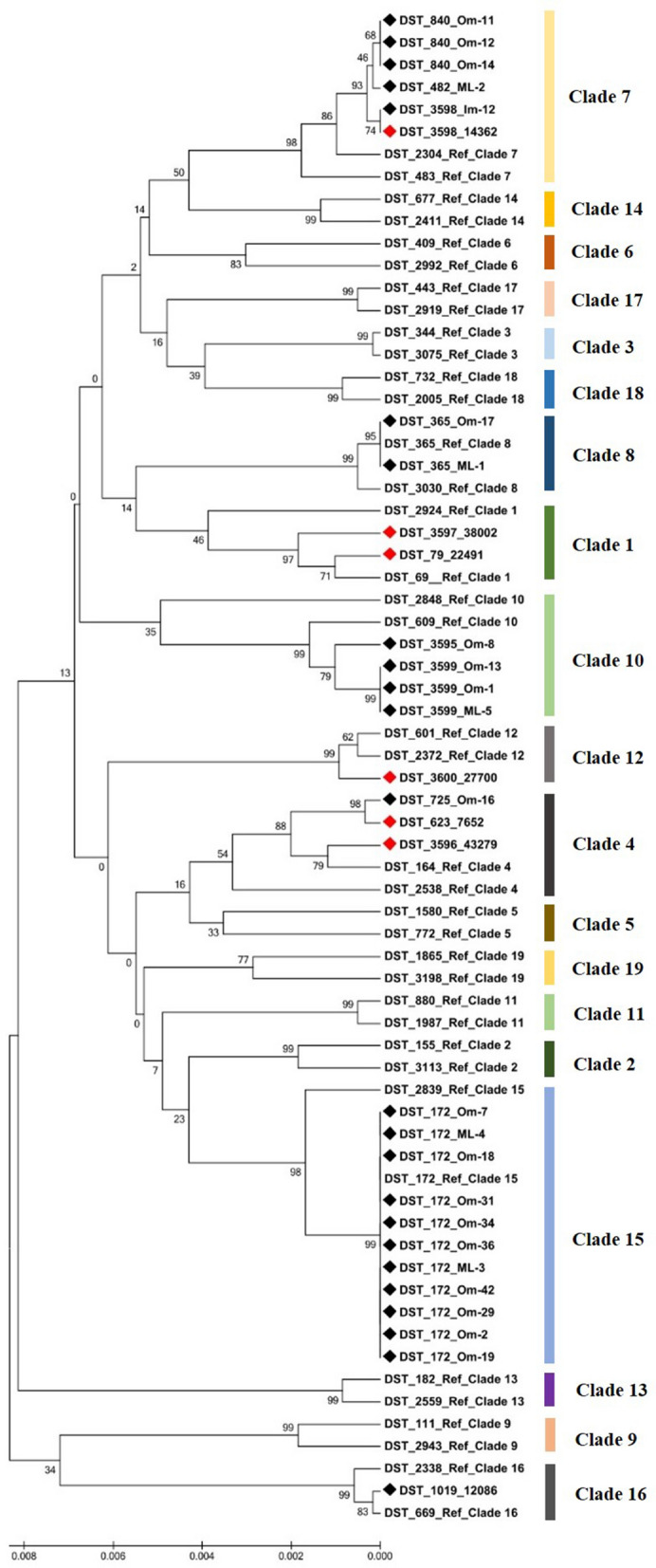
Evolutionary relationship of 30 *Candida albicans* isolates recovered in the study with reference isolates representing the MLST clades. The dendrogram was constructed by UPGMA analysis with p-distance method based on concatenated sequences of the seven loci. Black diamonds indicate isolates originated from animal source, while human isolates are highlighted with red diamonds. (Color figure online)

Fourteen unique DSTs were obtained with the combination of all seven allele numbers. Six of the 14 DSTs were new MLST genotypes (DST numbers: 3595–3600). Of note, 4 of the 6 new DSTs were recovered from human patients, whereas the majority of animal isolates belonged to previously described MLST genotypes (19/24; 79%) (Table 1). In order to reveal the phylogenetic relationship between isolates, we performed cluster analysis of isolates included in the present study and two reference DSTs from each previously assigned clades determined by UPGMA analysis [10]. The 30 *C. albicans* isolates grouped into 8 clades. The most prevalent genotype of bird isolates was DST 172 (11/24; 45.8%) which clustered into Clade 15. Clade 7 was the second most common clade (5/24; 20.8%), followed by Clade 10 (4/24; 16.6%), Clade 8 (2/24; 8.3%), Clade 4 (1/24; 4.2%) and Clade 16 (1/24; 4.2%). Human isolates grouped into Clade 1 (2/6; 33.3%), Clade 4 (2/6; 33.3%), Clade 7 (1/6; 16.6%) and Clade 12 (1/6; 16.6%). Interestingly, one waterfowl isolate (Om-16) and one human isolate (7652) was genetically closely related (Fig. 1). Moreover, the Im-12 isolate derived from ostrich and the human isolate 14362 shared the same DST, confirming that there is no host specificity of *C. albicans* strains in certain genotypes (Table 1, Fig. 1).

**Fig. 2:**
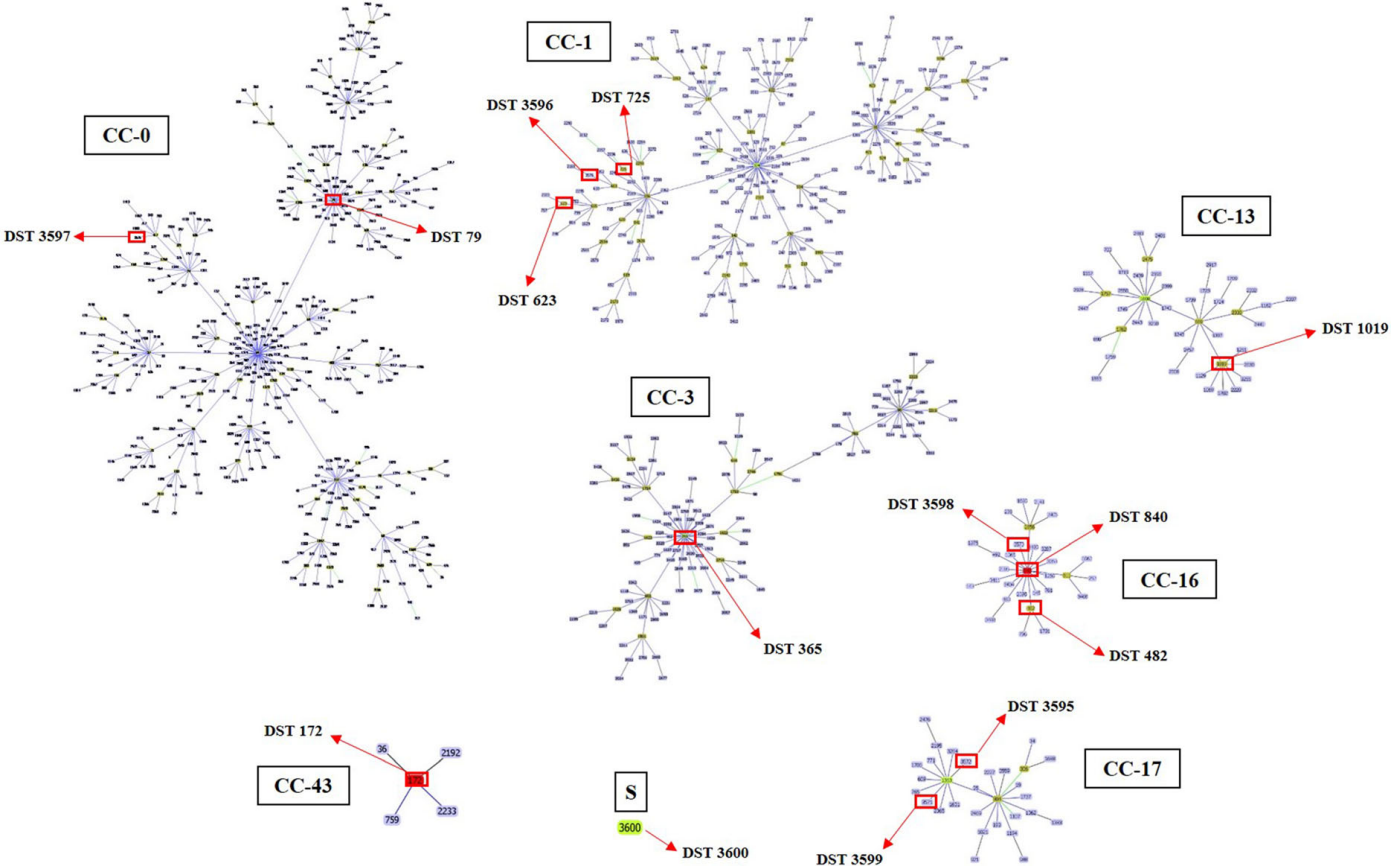
eBURST snapshot for *Candida albicans* diploid sequence types (DSTs) available in the MLST database. The illustration shows only those clonal complexes (CCs), which include Hungarian isolates (these are marked with red rectangles). A singleton (i.e. DST that could not be assigned to any group) is indicated with letter ‘S’. (Color figure online)

The allelic profiles of Hungarian *C. albicans* strains were compared with those deposited in the MLST database. Analysis of the genotypic relationship of strains using goeBURST algorithm yielded 3600 DSTs that were grouped into 156 CCs and 1113 singletons. CCs generated by goeBURST were arbitrarily numbered starting from 0 (for the CC with most DSTs). Clades determined by UPGMA corresponded well to the eBURST groups. The 30 isolates in this study were placed in 7 CCs, while one isolate was singleton (Table 1, Fig. 2). CC-43 contained 11 isolates of genotype DST 172, which was the putative founder of the group. DST 365 and DST 840 were also the predicted clonal founders of CC-3 and CC-16, respectively. The most abundant groups (CC-0, CC-1, CC-3) involved 7 isolates (3 bird and 4 human), whereas the remaining isolates were clustered to smaller groups as CC-13 (*n* = 1), CC-16 (*n* = 6) and CC-17 (*n* = 4). The newly identified animal isolates typed DST 3595 and DST 3599 belonged to CC-17 and both of them putatively evolved from DST 1363 group founder, which was identified from human samples according to the public database. The identical DST found among bird and human isolates, DST 3598 was assigned to CC-16 and probably developed from DST 840 group founder, like the closely related DST 482 (Fig. 2).

## Discussion

The MLST technique has contributed significantly to the understanding of the epidemiological and evolutionary relationships of different *C. albicans* strains due to its high discriminatory power [20]. We characterised the genetic diversity and population structure of *C. albicans* isolated from birds by the MLST method and assessed the genotypic distribution between hosts by involving human isolates as well. Based on nucleotide sequence variations in the seven housekeeping genes, 21 isolates were assigned eight previously known DSTs and nine isolates were assigned six new DSTs. Concerning human isolates the five major clades assigned by UPGMA (Clade 1, 2, 3, 4 and 11) have proved the most consistent over several years of rapid expansion of the MLST global database [7]. In our analysis, two human isolates (DST 623 and DST 3596) were assigned to Clade 4 and one bird isolate (DST 725). Two human isolates (DST 79 and DST 3597) clustered to Clade 1, while no isolate was found in Clade 2 and 11 suggesting that *C. albicans* isolates originating from animal source rather belong to minor clades. Furthermore, although Clade 1 is a major clade worldwide, it contains only a few isolates from animals raising the possibility that Clade 1 isolates may be better adapted to colonise and infect humans [21, 22].

MLST analysis revealed that isolates in the same eBURST clonal complexes were grouped together in the respective clades determined by UPGMA clustering, which was consistent with former observations [2, 23, 24]. None of the known DSTs found in our study were identified exclusively from animals (https://pubmlst.org/organisms/candida-albicans). New DSTs were recovered mainly from humans, suggesting that *C. albicans* is exposed to higher selective pressure in this host, which along with hospital environment may promote a more rapid evolution of this yeast. Taking the results into consideration, similarly to previous conclusion [11, 22] *C. albicans* subpopulations from birds and humans presumably develop relatively independently, while still maintaining some common features enabling the transfer of several genotypes between humans and animals.

To the best of our knowledge, the present study in the first to report molecular typing by MLST method and phylogenetic analysis of avian *C. albicans* isolated in Hungary. The low sample size and the lack of MLST genotyping of additional isolates from humans and other animals prevented us to perform extensive country-wide comparison of field isolates. However, we have gained insight into the molecular epidemiology and population evolution of *C. albicans* in birds. Considering that isolates from distinct genotypes or clades may have different phenotypes in terms of virulence, pathogenesis and drug resistance, our results provide good basis for further genome-based analyses.

## Data Availability

The ITS sequence data are available in the GenBank with accession numbers MT136511-MT136532 and MT478010-MT478017. Allele sequences and DST numbers of Hungarian *C. albicans* isolates are available in MLST database (https://pubmlst.org/organisms/candida-albicans).
